# Supplementary material for: Summer diatom blooms in the eastern North Pacific gyre investigated with a long-endurance autonomous surface vehicle
Source: PeerJ. 2018 Aug 15;6:e5387. doi: 10.7717/peerj.5387 (PMC6098680; doi:10.7717/peerj.5387)
Supplement: Supplemental Information 1 [file peerj-06-5387-s001.pdf]

Supplimental text

Biofouling was a factor which had to be accounted for in our analysis and was obvious later in the mission both on the on the downward-facing camera images (Fig. S1) and the Holo imagery. The copper tape in the sampling chamber did not eliminate the gradual buildup of a fine biofilm on the optics, although there were no filamentous algae or attached animals in the sample chamber (Fig. S2a). There was an extensive amount of corrosion on the camera arm of the Holo later traced to an incorrect metal-metal contact inside the housing. However, the corrosion did not extend into the optical path of the imaging (Fig. S2b,c) and is not believed to have contributed to the long-term average fouling. The Holo_Batch software was useful in filtering out particles which stayed in place through multiple holograms, such as algae or other particles adhered to the optical surfaces. Using the methodology described in the paper, we removed fixed particle averages using a particle averaging technique based on a 510 image bin. The software recognized location and size of particles from this average and subtracted it as a background from every image processed in batch mode. Holo_Detail did not require this correction as the particles of interest (*Hemiaulus* and *Rhizosolenia*) were visually recognized. We plotted the background value as percent of total biovolume removed to evaluate how the signal:noise ratio in the biovolume data changed over time (Fig. S3b). Around 24 June 2015, the percent of biovolume removed during processing increased from an average of 24.6% to an average of 53.8% and remained fairly steady until near the end of the mission (Fig. S3). Around 25 October 2015, the percentage removed steadily climbed to 97.3% on 2 November 2015. Although biofouling occurred, it appeared to reach a steady state before entering the waters around the Hawaiian Islands. Prior to 24 June 2015, it appeared that there was a sloughing off event which cleared the optical surfaces, resulting in the

decreased average removal percent of 24.6%. As the *Honey Badger* moved into the waters closer to the Hawaiian Islands, the biovolume steadily increased to around 2 μL L^{-1}, similar to the values observed in the *Hemiaulus* and *Rhizosolenia* blooms, 1.63 μL L^{-1} and 2.33 μL L^{-1}, respectively. The increase in biovolume and percent of biovolume removed may suggest that the biofouling had become so severe that most of the optical surfaces were covered or that the biofouling had produced filaments which were mobile enough to avoid removal by the averaging technique in the Holo_Batch software. In the raw holograms, these later images appear blurry with many very small diffraction patterns which do not come into focus using the Holo_Detail software (Fig S4). The corrosion layer noted in Fig. S2 would have completely blocked light resulting in dark images, not the observed numerous small refraction patterns.
