# Supplementary figures and images for: Summer diatom blooms in the eastern North Pacific gyre investigated with a long-endurance autonomous surface vehicle
Source: PeerJ. 2018 Aug 15;6:e5387. doi: 10.7717/peerj.5387 (PMC6098680; doi:10.7717/peerj.5387)

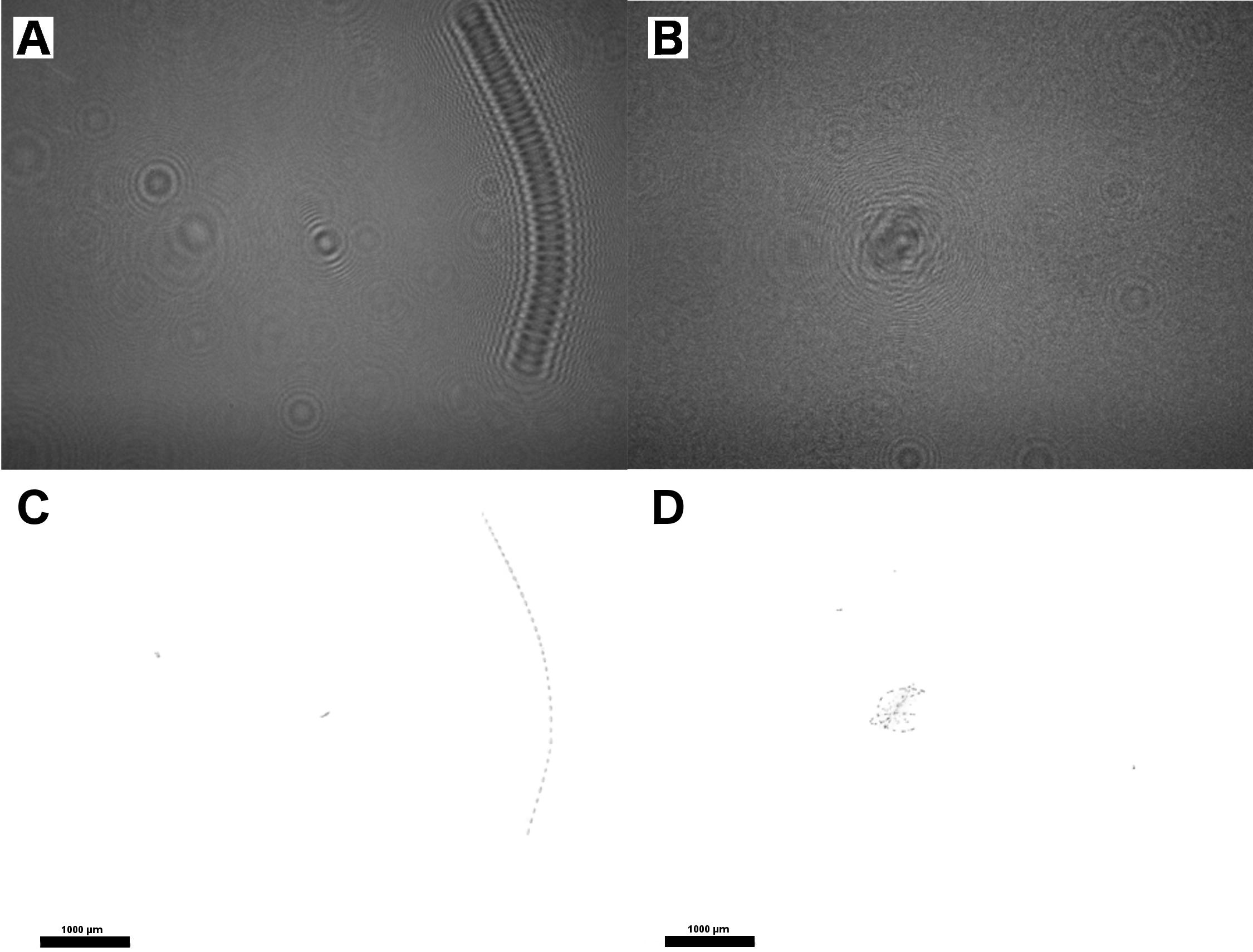

Supplement: Supplemental Information 2 — (A) clean hologram early in mission (image 004-0973; 6/14/15 2:54), (B) dirty hologram late in mission (image 004-9029), note the extensive background refraction patterns caused by fouling. (C) Batch processed (image 004-0973: 10/27/15 19:57) (D) Batch processed (image 004-0973; 6/14/15 02:54). [file peerj-06-5387-s002.png]

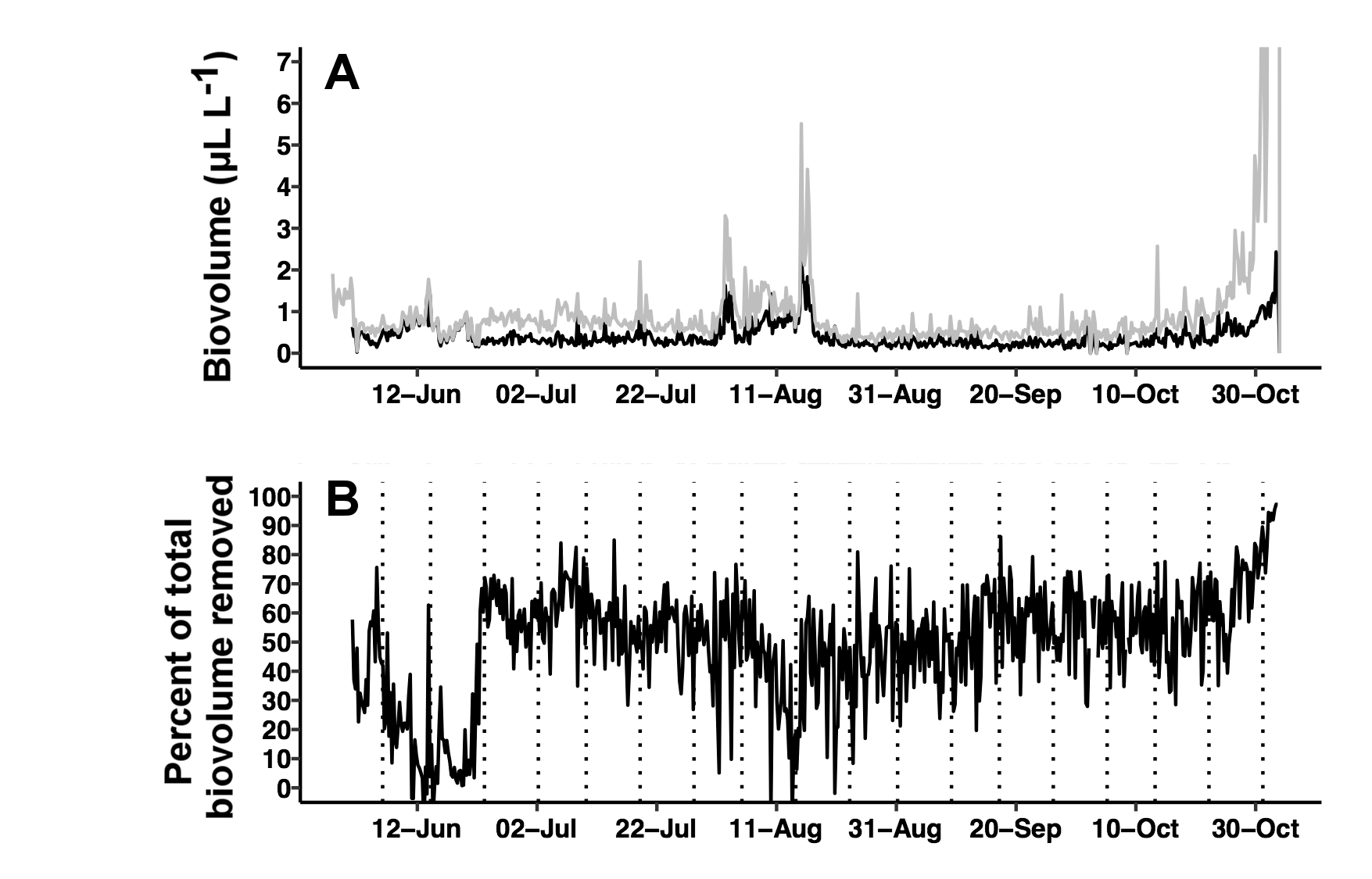

Supplement: Supplemental Information 3 — (A) Total biovolume reported by the LISST-Holo software (grey line) and corrected biovolume after background removal (black line). (B) Percentage of biovolume removed from the images over time. Vertical dotted lines are endpoints of the background averaging window (see text for details). [file peerj-06-5387-s003.png]

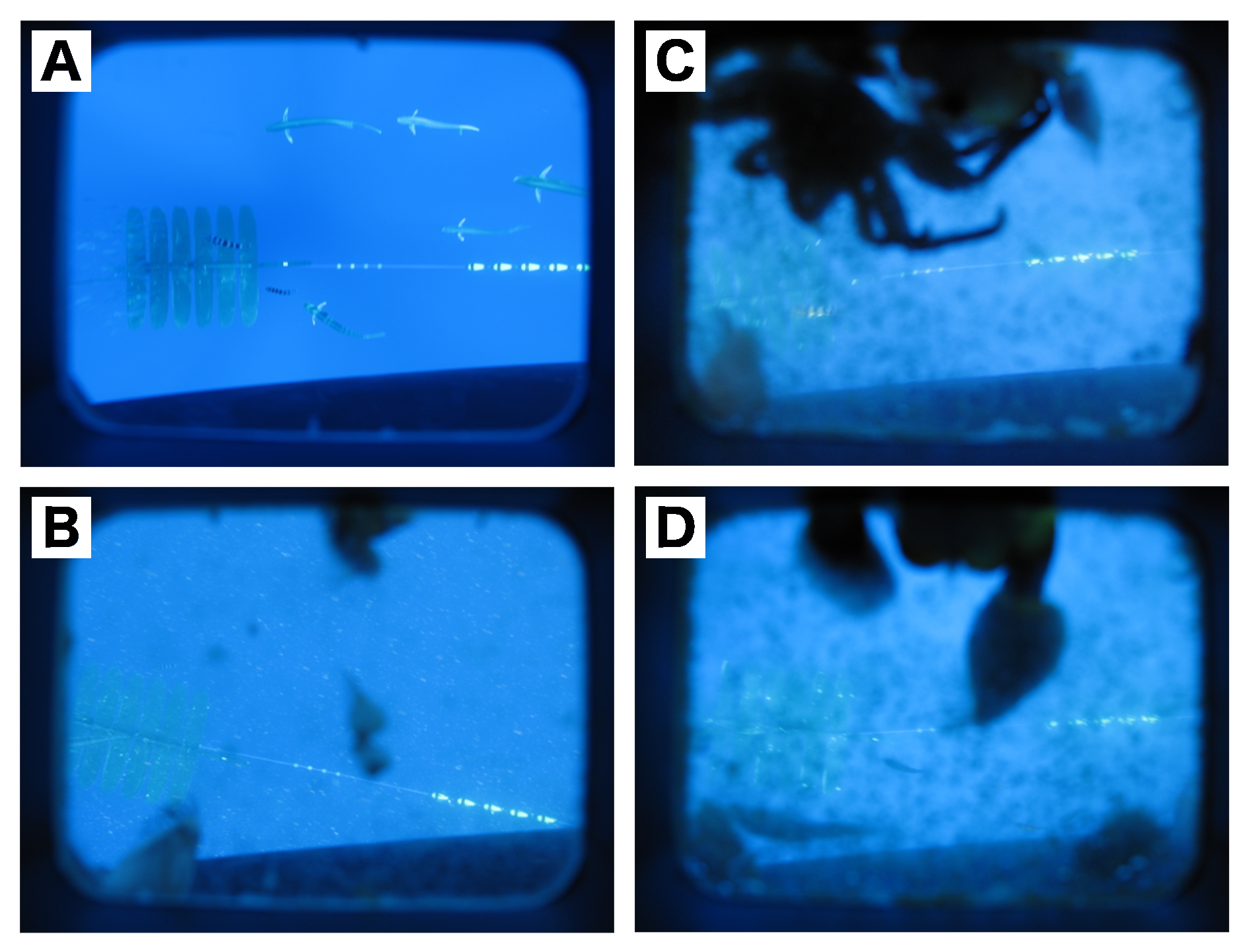

Supplement: Supplemental Information 4 — (A) IMG_20150701220016, 1 July 2015 1200 local time, (B) IMG_20150823220014, 23 Aug. 2015 1200 local time. This image illustrates an apparent accumulation of marine aggregates. (C) IMG 20150920170016. 21 Sept. 2015 0700 local time. (D) IMG_201510312200 31 Oct. 2015 1200 local time. [file peerj-06-5387-s004.png]

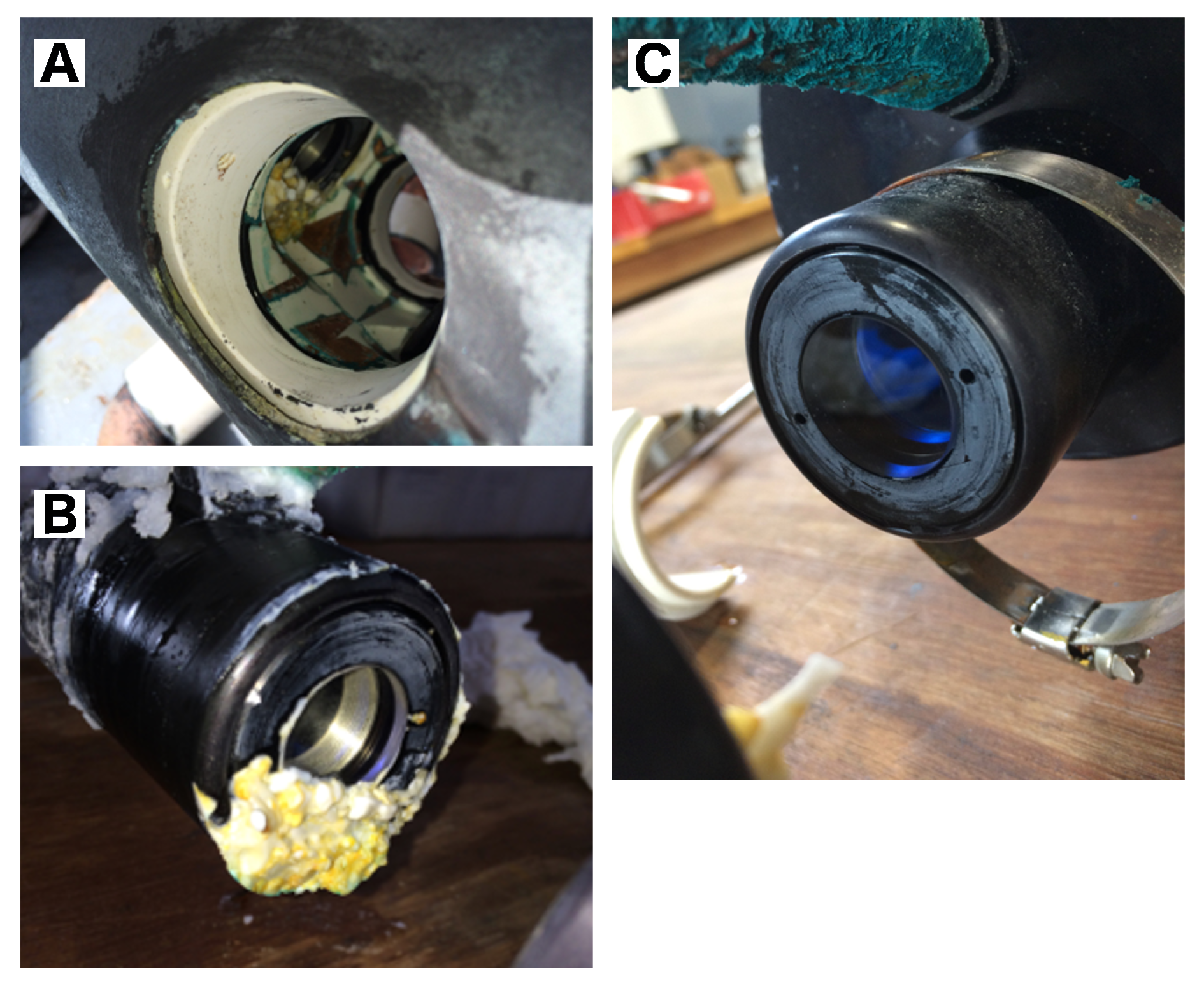

Supplement: Supplemental Information 5 — (A) Looking into the sampling chamber. Corrosion is evident as a yellow-white material on the lower left of the camera. Note that it does not extend into the active optical sampling area. No macroscopic fouling is evident. (B) close up of corrosion after LISST-Holo was removed from the tow body. (C) Laser optical window noting no corrosion or macroscopic fouling. [file peerj-06-5387-s005.png]

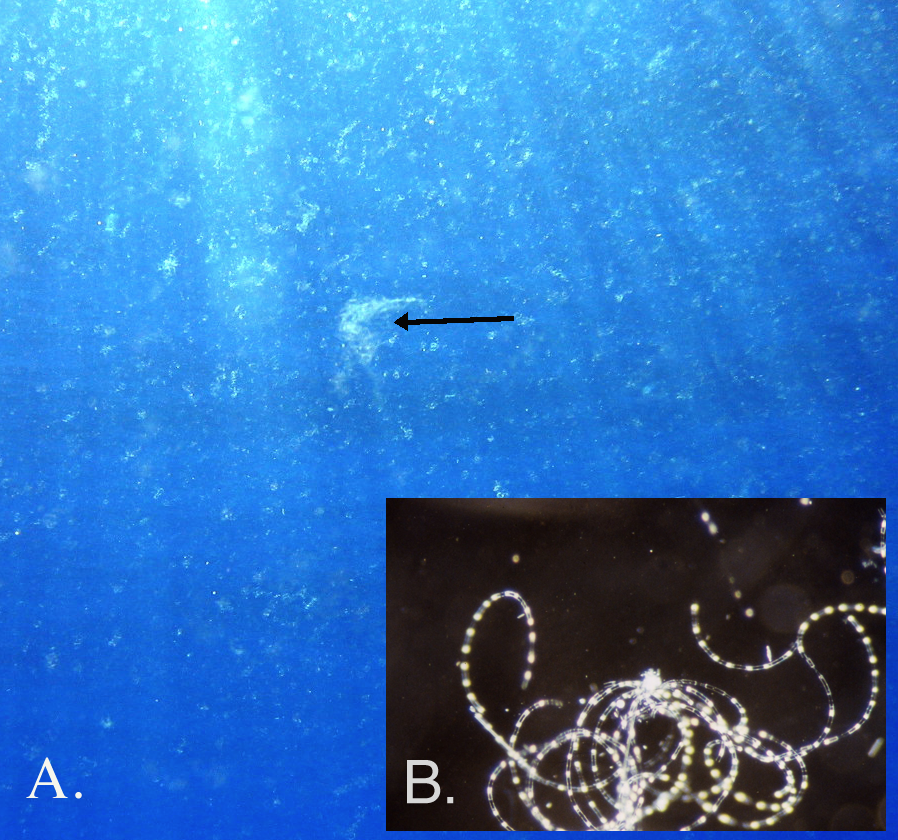

Supplement: Supplemental Information 6 — (A) The arrow indicates a Rhizosolenia mat. The remainder of the flocs were Hemiaulus aggregates (B) Stereoscope microgaphy of a Hemiaulus aggregate. Figure from Villareal et al. (2011). [file peerj-06-5387-s006.png]

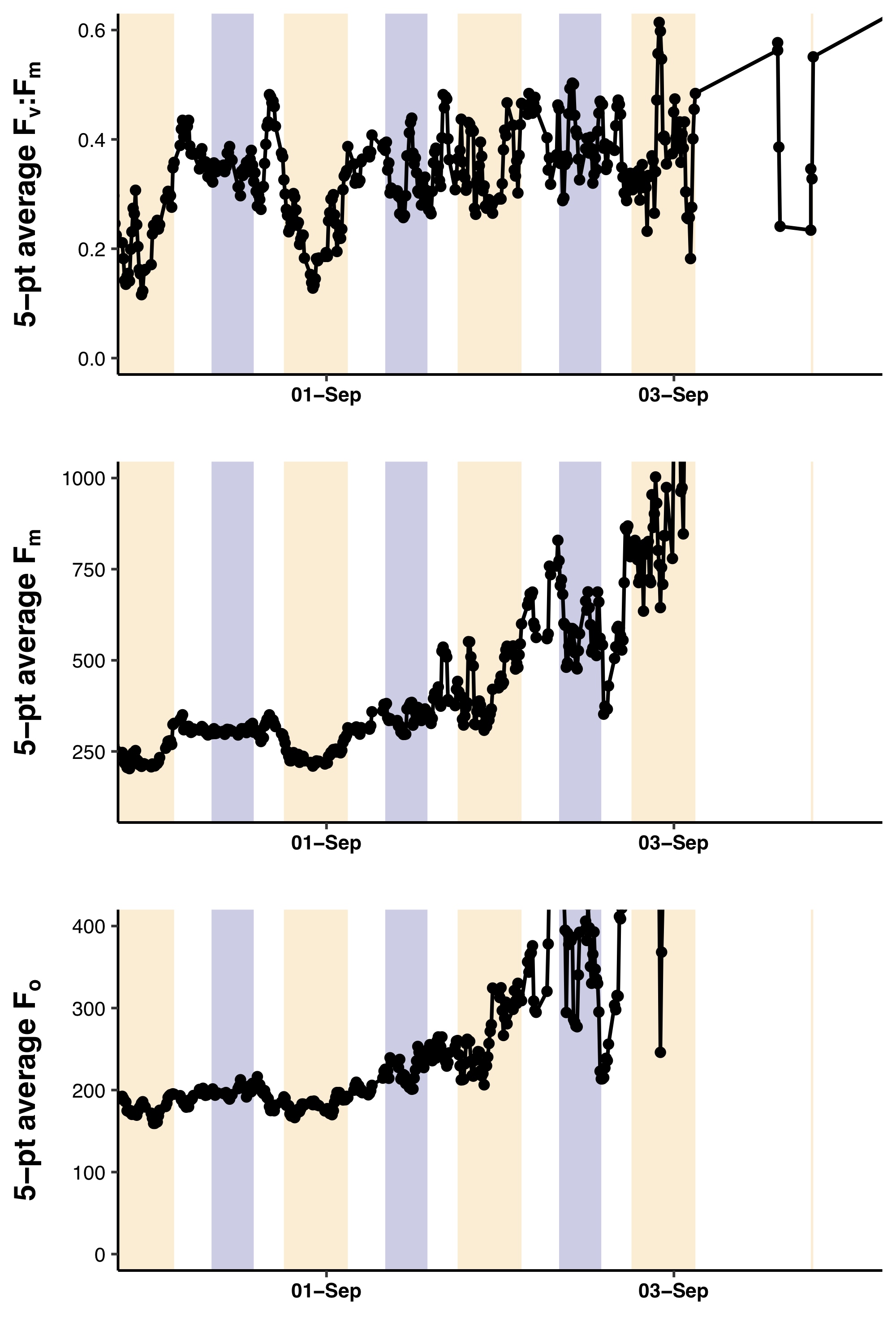

Supplement: Supplemental Information 7 — Fv:Fmvalues showed a normal diel rhythm (nocturnal maxima; diurnal minima) up to 1 September 2015. At this time, both the Fmand Fo used to calculated this value began a fatal upward drift that was not reflective of ambient chlorophyll concentrations from the satellite data. The values exceeded previously observed values by a factor of 3–4 until the instrument shut down and could not be restarted. Data after mid-day 1 Sept. is considered compromised and not useable for Fe-index determination. It is indicated on Fig. 9b as the asterisked points. [file peerj-06-5387-s007.png]
