# Supplementary material for: Summer diatom blooms in the eastern North Pacific gyre investigated with a long-endurance autonomous surface vehicle
Source: PeerJ. 2018 Aug 15;6:e5387. doi: 10.7717/peerj.5387 (PMC6098680; doi:10.7717/peerj.5387)
Supplement: Supplemental Information 8 [file peerj-06-5387-s008.pdf]

Supplemental information captions

Figure S1. Hologram fouling comparison (A) clean hologram early in mission (image 004-0973; 6/14/15 2:54), (B) dirty hologram late in mission (image 004-9029), note the extensive background refraction patterns caused by fouling. (C) Batch processed (image 004-0973: 10/27/15 19:57) (D) Batch processed (image 004-0973; 6/14/15 2:54)

Figure S2. Biofouling corrections and impact on volume calculations. (A) Total biovolume reported by the LISST-Holo software (grey line) and corrected biovolume after background removal (black line). (B) Percentage of biovolume removed from the images over time. Vertical dotted lines are endpoints of the background averaging window (see text for details).

Figure S3. Images from the Canon G10 downward facing camera illustrating biofouling buildup. A) IMG_20150701220016, 1 July 2015 1200 local time, (B) IMG_20150823220014, 23 Aug. 2015 1200 local time. This image illustrates an apparent accumulation of marine aggregates. (C) IMG 20150920170016. 21 Sept. 2015 0700 local time. (D) IMG_201510312200 31 Oct. 2015 1200 local time.

Figure. S4. LISST-Holo sampling chamber and camera. (A) Looking into the sampling chamber. Corrosion is evident as a yellow-white material on the lower left of the camera. Note that it does not extend into the active optical sampling area. No macroscopic fouling is evident. (B) close up of corrosion after LISST-Holo was removed from the tow body. (C) Laser optical window noting no corrosion or macroscopic fouling.

Figure S5. *Hemiaulus* snowstorm observed on 31 Aug. 2003 in the central N. Pacific gyre. (A) The arrow indicates a *Rhizosolenia* mat. The remainder of the flocs were *Hemiaulus* aggregates (B) Stereoscope micrograph of a *Hemiaulus* aggregate. Figure from Villareal et al (2011).

Figure S6. PhytoFlash diel rhythms immediately before the PhytoFlash failure in early September. F_v:F_m values showed a normal diel rhythm (nocturnal maxima; diurnal minima) up to 1 September 2015. At this time, both the F_m and F_o used to calculated this value began a fatal upward drift that was not reflective of ambient chlorophyll concentrations from the satellite dat(A) The values exceeded previously observed values by a factor of 3-4 until the instrument shut down and could not be restarted. (D) Data after mid-day 1 Sept. is considered compromised and not useable for Fe-index determination. It is indicated on Fig 9b as the asterisked points.
